# Xylanase increased the energetic contribution of fiber and improved the oxidative status, gut barrier integrity, and growth performance of growing pigs fed insoluble corn-based fiber

**DOI:** 10.1093/jas/skaa233

**Published:** 2020-07-20

**Authors:** Amy L Petry, Nichole F Huntley, Michael R Bedford, John F Patience

**Affiliations:** 1 Department of Animal Science, Iowa State University, Ames, IA; 2 AB Vista Feed Ingredients, Marlborough, Wiltshire, UK

**Keywords:** antioxidant capacity, arabinoxylan-oligosaccharide, corn bran, insoluble fiber, pig, xylanase

## Abstract

The experimental objective was to investigate the impact of xylanase on the bioavailability of energy, oxidative status, and gut function of growing pigs fed a diet high in insoluble fiber and given a longer adaptation time than typically reported. Three replicates of 20 gilts with an initial body weight (**BW**) of 25.43 ± 0.88 kg were blocked by BW, individually housed, and randomly assigned to one of four dietary treatments: a low-fiber control (**LF**) with 7.5% neutral detergent fiber (**NDF**), a 30% corn bran without solubles high-fiber control (**HF**; 21.9% NDF), HF + 100 mg/kg xylanase (**HF + XY**; Econase XT 25P), and HF + 50 mg/kg arabinoxylan-oligosaccharide (**HF + AX**). Gilts were fed ad libitum for 36 d across two dietary phases. Pigs and feeders were weighed on days 0, 14, 27, and 36. On day 36, pigs were housed in metabolism crates for a 10-d period, limit fed (80% of average ad libitum intake), and feces and urine were collected the last 72 h to determine the digestible energy (**DE**) and metabolizable energy (**ME**). On day 46, serum and ileal and colonic tissue were collected. Data were analyzed as a linear mixed model with block and replication as random effects, and treatment, time, and treatment × time as fixed effects. There was a significant treatment × time interaction for BW, average daily gain (**ADG**), and gain to feed (**G:F**; *P* < 0.001). By design, BW at day 0 did not differ; at day 14, pigs fed LF were 3.5% heavier, and pigs fed HF + XY, when compared with HF, were 4% and 4.2% heavier at days 27 and 36, respectively (*P* < 0.001). From day 14 to 27 and day 27 to 36, when compared with HF, HF + XY improved ADG by 12.4% and 10.7% and G:F by 13.8% and 8.8%, respectively (*P* < 0.05). Compared with LF, HF decreased DE and ME by 0.51 and 0.42 Mcal/kg, respectively, but xylanase partially mitigated that effect by increasing DE and ME by 0.15 and 0.12 Mcal/kg, over HF, respectively (*P* < 0.05). Pigs fed HF + XY had increased total antioxidant capacity in the serum and ileum (*P* < 0.05) and tended to have less circulating malondialdehyde (*P* = 0.098). Pigs fed LF had increased ileal villus height, and HF + XY and HF + AX had shallower intestinal crypts (*P* < 0.001). Pigs fed HF + XY had increased ileal messenger ribonucleic acid abundance of claudin 4 and occludin (*P* < 0.05). Xylanase, but not AX, improved the growth performance of pigs fed insoluble corn-based fiber. This was likely a result of the observed increase in ME, improved antioxidant capacity, and enhanced gut barrier integrity, but it may require increased adaptation time to elicit this response.

## Introduction

Dietary energy impacts nearly every performance metric in pork production (Beaulieu et al., 2009), and meeting the specification for dietary energy accounts for more than 60% of the input cost of raising one hog to market (Patience, 2017). One approach to reduce this cost is to improve the energetic contribution of dietary components that supply energy: protein, fat, simple carbohydrates, and fiber. Arguably, of these four, fiber has the greatest opportunity for improvement. Typically, swine diets in the United States contain between 8% and 20% fiber, generally insoluble and corn-based, and within these diets, the contribution of fiber to energy is estimated to be less than 4%, irrespective of the energy system (Patience and Petry, 2019). One strategy to improve the energetic contribution of fiber is to include carbohydrases, such as xylanase, into the formulation matrix. Xylanase hydrolyzes the β-(1-4) glycosidic bonds of arabinoxylan by releasing a mixture of xylose, arabinose, and xylooligosaccharides that can be either absorbed or fermented by the pig (Dodd and Cann, 2009), but its efficacy in corn-based diets is inconsistent and poorly understood.

In theory, supplementing xylanase in diets with corn-based fiber, a fiber source rich in arabinoxylan, should improve the energetic contribution of that fiber resulting in improved growth performance. Indeed, studies have shown improvements in both fiber and energy digestibility when supplementing corn coproducts with xylanase, but few studies report both an improvement in digestibility and performance (Torres-Pitarch et al., 2019). It is poorly understood why these responses occur, or lack thereof, but they may be attributed to the length of xylanase supplementation time or the concentration of fiber within the diet. It has been reported that improvements in fiber and energy digestibility in the upper small intestine required at least 25 d of adaptation to xylanase (Petry et al., 2020)— a period of time significantly longer than adaptation times typically reported within the literature. Furthermore, in recent years, the role of xylanase has extended beyond its original intent due to reports of unexpected health benefits, particularly reductions in mortality in grow-finish pig production (Zier-Rush et al., 2016). There have also been reports of improvements in gut barrier integrity (Tiwari et al., 2018) and reductions in markers of oxidative stress (Duarte et al., 2019) in nursery pigs supplemented with xylanase, but there is a dearth of literature in the grow-finish pig. Therefore, the experimental objective was to investigate the impact of xylanase on energy bioavailability, oxidative status, and gut function and morphology of growing pigs fed a diet higher in insoluble fiber and given a longer adaptation time than typically reported. It was hypothesized that when given sufficient adaptation time, xylanase would improve the energetic contribution of corn-based fiber, growth performance, markers of gut barrier integrity, and oxidative status of growing pigs.

## Materials and Methods

Experimental procedures reported herein adhered to guidelines for the ethical and humane use of animals for research according to the *Guide for the care and use of agricultural animals in research and teaching* (FASS, 2010) and were approved by the Iowa State University Institutional Animal Care and Use Committee (#9-17-8613-S).

### Animals, housing, and experimental design

Sixty crossbred gilts (L337 × Camborough; PIC Inc., Hendersonville, TN) with an initial body weight (**BW**) of 25.4 ± 0.9 kg were used in three replicates of a 46-d trial. Twenty gilts were included in each replicate and the same procedures were applied across replicates. Pigs were blocked by initial BW and randomly assigned within a block to one of four dietary treatments. Pigs were individually housed for 36 d in pens (1.8 ×. 1 m) equipped with a partially slatted concrete floor, an automatic self-feeder, and a cup drinker. On day 36, pigs were moved to metabolism crates (1.50 × 0.7 m) for 10 d in total to facilitate 7 d of adaptation followed by 72 h of fecal and urine collections. The metabolism crates allowed for separate collection of feces and urine and were equipped with a slatted floor, feeder, and nipple waterer. Pigs were necropsied at experimental termination for intestinal tissue collection.

### Diets and feeding

Four dietary treatments were evaluated: a low-fiber control (**LF**) with 7.5% neutral detergent fiber (**NDF**), a 30% corn bran without solubles higher-fiber control (**HF**; NDF = 21.9%), HF + 100 mg xylanase/kg (**HF + XY**; Econase XT 25P; AB Vista, Marlborough, UK) providing 16,000 birch xylan units per kilogram, and HF + 50 mg arabinoxylan-oligosaccharide/kg (**HF + AX**; 3 to 7 degrees of polymerization). All diets were formulated to meet or exceed the National Research Council (NRC, 2012) requirements for growing pigs and were manufactured in mash form. During mixing, 10 representative samples of each diet were randomly collected throughout the mixing batch, homogenized, and stored at −20 °C for future analysis. Chromium trioxide (Cr_2_O_3_) was included in phase 2 diets as an indigestible marker to determine the energy digestibility during the metabolism crate period. Pigs were fed ad libitum for 36 d across two dietary phases: phase 1: from day 0 to 27 and phase 2: from day 27 to 36 (Tables 1 and 2). From day 36 to 46, pigs were limit fed 80% of the average daily feed intake (**ADFI**) among all treatments of the first replicate, and the same feed allotment was used in subsequent replicates. When limit fed, the daily feed allotment was split into two feedings at 0700 and 1500 hours. Any orts remaining after 1 h were collected, dried, and weighed. Pigs had ad libitum access to water throughout the entire trial.

**Table 1.** Ingredient and nutrient composition of experimental diets (as-fed basis): phase 1^1^

	Treatment^2^			
Item	LF	HF	HF + XY	HF + AX
Ingredient composition, %				
Corn	74.548	44.526	44.516	44.521
Corn bran without solubles	0.000	30.000	30.000	30.000
Soybean meal	22.347	22.347	22.347	22.347
Limestone	1.231	1.207	1.207	1.207
Monocalcium phosphate 21%	0.569	0.657	0.657	0.657
Sodium	0.500	0.500	0.500	0.500
l-lysine HCL	0.322	0.293	0.293	0.293
Trace Mineral Premix	0.200	0.200	0.200	0.200
Vitamin Premix^2^	0.140	0.140	0.140	0.140
l-threonine^3^	0.078	0.064	0.064	0.064
dl-methionine	0.062	0.059	0.059	0.059
Quantum Blue, 5G	0.005	0.005	0.005	0.005
Econase 25 P	0.000	0.000	0.010	0.000
AX^5^	0.000	0.000	0.000	0.005
Calculated nutrients				
SID Lysine, %	0.98	0.98	0.98	0.98
SID TSAA:Lysine	0.56	0.56	0.56	0.56
SID Threonine: Lysine	0.60	0.60	0.60	0.60
SID Trpytophan:Lysine	0.17	0.17	0.17	0.17
Ca, %	0.66	0.66	0.66	0.66
STTD P, %	0.33	0.33	0.33	0.33
ME, Mcal/kg	3.28	3.02	3.02	3.02
NE, Mcal/kg	2.46	2.25	2.25	2.25

^1^Phase 1 was fed from day 0 to 27. ^2^LF, low-fiber control; HF, high-fiber control; HF+XY, high-fiber control containing 100 mg xylanase/kg; HF+AX, high-fiber control containing 50 mg arabinoxylanoligosaccharide/kg. ^3^Vitamin premix provided the following (per kg diet): 6,125 IU of vitamin A, 700 IU of vitamin D3, 50 IU of vitamin E, 3 mg of menadione (to provide vitamin K), 11 mg of riboflavin, 27 mg of d-pantothenic acid, 0.05mg of vitamin B12, and 56 mg of niacin. ^4^Mineral premix provided the following (per kg diet): 165 mg of Fe (ferrous sulfate), 165 mg of Zn (zinc sulfate), 39 mg of Mn (manganese sulfate), 16.5 mg of Cu (copper sulfate), 0.3 mg of I (calcium iodate), and 0.3 mg of Se (sodium selenite). ^5^3 to 7 degrees of polymerization.

**Table 2.** Ingredient and nutrient composition of experimental diets (as-fed basis): phase 2^1^

	Treatment^2^			
Item	LF	HF	HF + XY	HF + AX
Ingredient composition, %				
Corn	75.996	45.975	45.965	45.970
Corn bran without solubles	0.000	30.000	30.000	30.000
Soybean meal	20.566	20.566	20.566	20.566
Limestone	1.182	1.159	1.159	1.159
Monocalcium phosphate 21%	0.504	0.593	0.593	0.593
Sodium	0.500	0.500	0.500	0.500
Cr_2_O_3_	0.500	0.500	0.500	0.500
l-lysine HCL	0.295	0.267	0.267	0.267
Trace mineral premix^3^	0.200	0.200	0.200	0.200
Vitamin premix^2^	0.140	0.140	0.140	0.140
l-threonine	0.067	0.054	0.054	0.054
dl-methionine	0.043	0.041	0.041	0.041
Quantum Blue, 5G	0.005	0.005	0.005	0.005
Econase 25 P	0.000	0.000	0.010	0.000
AX^5^	0.000	0.000	0.000	0.005
Calculated nutrients				
SID Lysine, %	0.92	0.92	0.92	0.92
SID TSAA:Lysine	0.56	0.56	0.56	0.56
SID Threonine: Lysine	0.61	0.61	0.61	0.61
SID Trpytophan:Lysine	0.17	0.17	0.17	0.17
Ca, %	0.63	0.63	0.63	0.63
STTD P, %	0.31	0.31	0.31	0.31
ME, Mcal/kg	3.26	3.02	3.02	3.02
NE, Mcal/kg	2.46	2.26	2.26	2.26
Analyzed composition,%				
DM	89.12	89.81	89.98	90.15
Starch	41.08	23.55	23.70	23.11
CP	15.56	15.54	15.47	15.60
NDF	7.54	21.91	22.12	22.08
ADF	2.41	5.53	5.78	5.59
aEE	2.48	2.74	2.82	2.89
Cr_2_O_3_	0.49	0.49	0.49	0.49

^1^Phase 2 diets were fed from day 27 to 46. ^2^LF, low-fiber control; HF, high-fiber control; HF+XY, high-fiber control containing 100 mg xylanase/kg; HF+AX, high-fiber control containing 50 mg arabinoxylanoligosaccharide/kg. ^3^Vitamin premix provided the following (per kg diet): 6,125 IU of vitamin A, 700 IU of vitamin D3, 50 IU of vitamin E, 3 mg of menadione (to provide vitamin K), 11 mg of riboflavin, 27 mg of d-pantothenic acid, 0.05mg of vitamin B12, and 56 mg of niacin. ^4^Mineral premix provided the following (per kg diet): 165 mg of Fe (ferrous sulfate), 165 mg of Zn (zinc sulfate), 39 mg of Mn (manganese sulfate), 16.5 mg of Cu (copper sulfate), 0.3 mg of I (calcium iodate), and 0.3 mg of Se (sodium selenite). ^5^3 to 7 degrees of polymerization.

### Sample and data collection

During the adaptation period, pigs and feeders were weighed on days 0, 14, 27, and 36 to calculate average daily gain (**ADG**), ADFI, and feed efficiency (**G:F**). On day 0, blood was collected via jugular venipuncture into a vacutainer tube (Becton Dickinson, Franklin Lakes, NJ) and centrifuged at 1,500 × *g* for 15 min at 4 °C. The resulting serum was stored at −80 °C for later baseline analysis. During the last 72 h of each replicate, total urine and fresh fecal grab samples were collected at 0730 and 1530 hours each day. Urine was collected in acid-washed 4-liter bottles containing 6 M HCl to ensure that the pH was maintained below 2.0 to minimize nitrogen volatilization. Urine was weighed, filtered through glass wool, subsampled, and stored in acid-washed plastic containers at −20 °C until further analysis. Fecal samples were amassed, homogenized, and immediately stored at −20 °C.

On day 46, after 0730 hours urine and fecal collections and prior to euthanizing, pigs were fed half their total daily feed allotment, weighed, and blood was collected via jugular venipuncture into two 10 mL tubes for plasma and serum. Plasma and serum were separated by centrifugation (2,000 × *g* for 15 min at 4 °C and 1,500 × *g* for 15 min at 4 °C, respectively), collected, subsampled, and stored at −80 °C until analyzed. The pigs were then euthanized by captive bolt stunning and exsanguination. A 24-cm section of ileal tissue was collected 20 cm proximal to the ileocecal junction and rinsed with phosphate-buffered solution. From the isolated section, two 6-cm sections were snap-frozen in liquid nitrogen and stored at −80 °C for later analysis, and two 6-cm sections were fixed in 10% neutral-buffered formalin. A 6-cm section proximal to the ascending spiral colon was removed, flushed with phosphate-buffered solution, snap-frozen in liquid nitrogen, and stored at −80 °C for later analysis.

### Diet, urine, and fecal analytical methods

At the conclusion of each replicate, urine and fecal samples were thawed, homogenized for each pig, and subsampled. Diet and fecal subsamples were dried in a convection oven at 60 °C until a constant weight was achieved and were ground to a particle size of 1.0 mm (Wiley Mill 3379-K35, Thomas Scientific, Swedesboro, NJ). Urine subsamples were thawed, mixed, and filtered through Whatman 41 filter paper (GE Healthcare Life Sciences, Chicago, IL, USA) prior to analysis.

Diets were analyzed in duplicate for acid-hydrolyzed ether extract (**aEE**; method 2003.06; AOAC, 2007) and nitrogen (method 990.03; AOAC, 2007; TruMac; LECO Corp., St. Joseph, MI). An ethylenediaminetetraacetate sample (9.56% nitrogen; determined to have 9.56 ± 0.08% nitrogen) was used for standard calibration and crude protein (**CP**) was calculated as nitrogen × 6.25. Diets were analyzed for starch using a Megazyme total starch assay kit (Wicklow, Ireland; modified method 996.11, AOAC, 1996). Diets were analyzed in triplicate for NDF using the method of Van Soest and Robertson (1979) and acid detergent fiber (**ADF**) according to Goering and Van Soest (1970).

Diet and fecal samples were analyzed in duplicate for dry matter (**DM**; method 930.15) and Cr_2_O_3_ using the method of Fenton and Fenton (1979). Diet and fecal samples were analyzed in duplicate for gross energy (**GE**) using a bomb calorimeter (model 6200; Parr Instrument Co., Moline, IL). Benzoic acid (6,318 kcal/kg; Parr Instrument Co.) was used as the standard for calibration and was determined to contain 6,322 ± 0.91 kcal/kg. For urine energy determination, 1.5 mL of urine was added to 0.25 g of cotton and subsequently freeze-dried for 48 h. Urine plus cotton dried samples were analyzed for GE in triplicate. Urinary energy was calculated from the difference of energy determined in cotton alone and the energy determined in the samples containing both urine and cotton. A coefficient of variation (CV) threshold of less than 1% was used for DM, Cr_2_O_3_, CP, and GE, and less than 3% for NDF, ADF, and aEE. 

### Messenger ribonucleic acid abundance and lipopolysaccharide-binding protein

Ileal tissue total ribonucleic acid (**RNA**) was extracted using a commercial kit (RNeasy Plus Mini Kit, Qiagen, Carlsbad, CA) and Qiagen Tissuelyser II (Germantown, MD). The concentration of RNA was quantified using a spectrophotometer (ND-100; NanoDrop Technologies Inc., Rockland, DE), and all samples had 260:280 nm ratios above 1.84. Complementary deoxyribose nucleic acid (**cDNA**) was transcribed from 0.8 μg RNA using the QuantiTect Reverse Transcription Kit (Qiagen, Hilden, Germany) and diluted 10-fold with nuclease-free water. 

Real-time quantitative polymerase chain reaction was performed using iQ SYBR Green Supermix (Bio-Rad Laboratories, Inc., Hercules, CA) in triplicate. The gene-specific primers (Table 3) were diluted to 10 µM with nuclease-free water. Each 20 µL reaction included 10 µL of SYBR Green Supermix, 1 µL of each forward and reverse primer, 3 µL of cDNA, and 5 µL of nuclease-free water. A pooled reference sample and a no-reverse transcriptase negative control were included for each gene. The SYBR Green fluorescence was quantified using a real-time polymerase chain reaction detection system (iQ5; Bio-Rad Laboratories Inc.). Cycling conditions were as follows: 5-min initial denaturation at 95 °C followed by 40 polymerase chain reaction cycles (95 °C for 30 s, 55 or 60 °C for 30 s, and 72 °C for 30 s) and a dissociation curve to verify the amplification of a single polymerase chain reaction product. Optical System Software (iQ5, version 2.0; Bio-Rad Laboratories Inc.) was used to analyze amplification plots and cycle threshold (**Ct**) values for each reaction were obtained. The messenger RNA (**mRNA**) abundance was normalized to the pooled sample and ribosomal protein L19 reference gene. A CV threshold of less than 5% was used. To calculate the fold change, the 2^−ΔΔCt^ method described by Livak and Schmittgen (2001) was implemented. Plasma lipopolysaccharide-binding protein (**LBP**) was measured with a commercially available enzyme-linked immunosorbent assay kit (Hycult Biotech, Plymouth Meeting, PA), and an inter-assay CV threshold of 5% was used.

**Table 3.** Primers used for real-time quantitative polymerase chain reaction

Gene	Primer sequence, 5′→3′ ^1^	Product size, bp	GenBank accession	Annealing Temperature, °C
*Claudin-3*	F: TTGCATCCGAGACCAGTCC	85	NM_001160075	60
	R: AGCTGGGGAGGGTGACA			
*Claudin-4*	F: CAACTGCGTGGATGATGAGA	140	NM_001161637	60
	R: CCAGGGGATTGTAGAAGTCG			
*Occludin*	F: TCGTCCAACGGGAAAGTGAA	95	NM_001163647	55
	R: ATCAGTGGAAGTTCCTGAACCA			
*zonula occuludens-1 (ZO-1)*	F: CTCTTGGCTTGCTATTCG	197	XM_003353439	55
	R: AGTCTTCCCTGCTCTTGC			
*ribosomal protein L19 (RPL19)*	F: AACTCCCGTCAGCAGATCC	147	AF_435591	55
	R: AGTACCCTTCCGCTTACCG			

^1^F, forward primer; R, reverse primer.

### Intestinal morphology

Fixed ileal tissues were embedded in paraffin wax, sectioned, stained with hematoxylin and eosin, and mounted on glass slides (Iowa State University Veterinary Diagnostic Lab, Ames, IA). Images of the slides were taken at 10× power using a DP80 Olympus Camera mounted on an OLYMPUS BX 53/43 microscope (Olympus Scientific, Waltham, MA). A total of 10 villi and crypt pairs across two ileal sample cross sections were measured using OLYMPUS CellSens Dimension 1.16 software. The measurements of the 10 villi and crypts were averaged and the ratio of villus height to crypt depth was calculated (**V:C**).

### Oxidative status measures

Malondialdehyde (**MDA**) was measured in serum and ileal and colonic tissue lysates using a thiobarbituric acid reactive substances kit (TBARS Assay Kit, Cayman Chemical Company, Ann Arbor, MI). The total antioxidant capacity (**TAC**) of serum and ileal and colonic tissue lysates were measured using a commercially available colorimetric assay (Antioxidant Assay Kit, Cayman Chemical Company, Ann Arbor, MI). Serum was diluted 1:100 with provided assay buffer, and all tissue lysates were normalized to a similar protein concentration. TAC reported herein was defined as the concentration of antioxidants within a sample that inhibited the oxidation of 2,2′-Azino-di-3-ethylbenzothiazoline sulfonate by metmyoglobin relative to a water-soluble tocopherol analog, Trolox, and quantified as millimolar Trolox equivalent (Miller et al., 1993). A CV threshold of less than 5% was used for MDA and TAC values.

### Calculations and statistical analysis

Digestible energy (**DE**) was calculated by multiplying GE intake by the total tract digestibility coefficient calculated according to the methods described by Oresanya et al. (2007). Metabolizable energy (**ME**) was calculated by subtracting urinary energy from DE; calculation of methane losses was omitted. Net energy (**NE**) was estimated from ME using the Noblet (1994) equation:

$$\begin{array}{ll} NE\;= & \;[0.726\times ME]+[1.33\;\times \;EE]\;\\ & +[0.39\times starch]\;\;[0.62\;\times CP]\;[0.83\;\times ADF] \end{array}$$

Data with an independent covariance structure were analyzed according to the following mixed model:

$$\begin{array}{l} Y_{ijkl}=\;\mu +\tau_{i\;}+\upsilon_{j}+\rho_{k\;}+e_{ijkl} \end{array}$$

Where $Y_{ijkl}$ is the observed value for lth experimental unit within the ith level of dietary treatment of the jth block for the lth pig in the kth replicate; μ is the general mean; $\tau_{i\;}$ is the fixed effect of the ith diet (i = 1 to 4); $\upsilon_{j}$ is the random effect of the jth block (j = 1 to 5); $\rho_{k\;}$ is the random effect of the kth replicate (k = 1 to 3); and $e_{ijkl}$ is the associated variance as described by the model for $Y_{ijkl}$ (l = 1 through 60); assuming $\upsilon_{j}\;∼N\left(0,\;I\sigma_{\upsilon_{j}}^{2}\right),\;\rho_{k\;}\;∼N(0,\;I\sigma_{\rho_{k\;}}^{2}$), and $e_{ijkl}\;∼N(0,\;I\sigma_{e}^{2}$), where *I* is the identity matrix.

Data that were collected at multiple time points were analyzed with a dependent covariance structure according to the following mixed model:

$$\begin{array}{l} Y_{ijklm}=\;\mu +\tau_{i\;}+\upsilon_{j}+\;\tau_{i\;}\upsilon_{j}+\rho_{k\;}+a_{l}+e_{ijklm} \end{array}$$

Where $Y_{ijklm}$ is the observed value for mth experimental unit at a given jth point in time within the ith level of dietary treatment of the lth block for the mth pig in the kth replicate; μ is the general mean; $\tau_{i\;}$ is the fixed effect of the ith diet (i = 1 to 4); $\upsilon_{j}$ is the fixed effect of time at a given jth measurement (j = 1 to 5); $\;\tau_{i\;}\upsilon_{j}$ is the interaction term for dietary treatment and time; $\rho_{k\;}$is the random effect of the kth replicate (k = 1 to 3); $a_{l}$ is the random effect of the lth block (l = 1 to 4); and $e_{ijkl}$ is the associated variance as described by the model for $Y_{ijkl}$ (l = 1 through 60); assuming $\upsilon_{j}\;∼N\left(0,\;AR1\right),\;\rho_{k}∼N(0,\;I\sigma_{\rho_{k\;}}^{2}$), $a_{l}\;∼N(0,\;I\sigma_{\alpha_{l}}^{2}$), and $e_{ijkl}∼N(0,\;I\sigma_{e}^{2}$), where *I* is the identity matrix and *AR1* is a first-order autoregressive covariance structure.

The PROC UNIVARIATE procedure in SAS 9.3 (SAS Inst., Cary, NC) was used to verify normality and homogeneity of the studentized residuals from the reported models. Each model was analyzed using PROC MIXED. Least square means were separated using Fisher’s least significant difference test, and treatment differences were considered significant if *P*≤ 0.05 and trends if 0.05 > *P*≤ 0.10.

## Results

All pigs within each replicate completed the trial. A few instances of diarrhea were observed prior to the metabolism period in replicates two and three. Pigs were treated with tylosin phosphate; whenever affected pigs were treated, all pigs in that replicate were treated as well. There were no significant interactions among replicate and other main effects for the reported dependent variables; therefore, the effect of replicate was included as a random effect within the respective models to account for this additional variance.

### Growth performance

**Figure 1.**
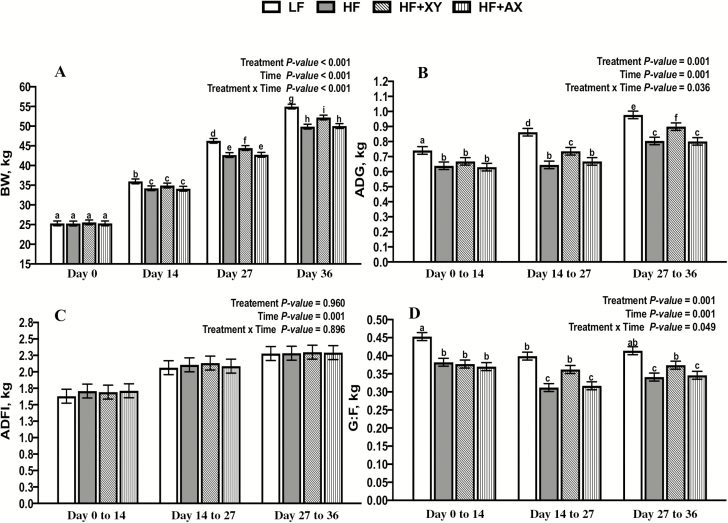
The impact of treatment, time, and treatment by time on the BW (A), ADG (B), ADFI (C), and G:F (D), of growing pigs fed insoluble corn-based fiber.

There was a significant interaction between treatment and time for BW (Treatment ×. Time *P* < 0.001; Figure 1A). Whereas, by design, BW at day 0 did not differ among treatments, at day 14, pigs in the LF treatment were 3.5% heavier, and pigs fed xylanase, when compared with HF, were 4% and 4.2% heavier at days 27 and 36, respectively (*P* < 0.05). Similarly, there was an interaction between treatment and time for ADG (Treatment ×. Time *P* = 0.036; Figure 1B). From day 0 to 14, LF gained 12.8% more per day among treatments, but from day 14 to 27 and day 27 to 36, HF + XY performed intermediately among treatments; when compared with HF, the addition of xylanase improved ADG by 12.4% and 10.7%, from day 14 to 27 and day 27 to 36, respectively (*P* < 0.05). ADFI did not differ among treatments across periods (Treatment *P* = 0.946; Figure 1C), but as time increased, ADFI increased as well (Time *P* = 0.001). This drove a time by treatment interaction for G:F (Treatment × Time *P* = 0.049; Figure 1D). In all weigh periods, LF had the greatest feed efficiency, and the addition of 30% corn bran without solubles reduced G:F by 15.7 %, 21.8%, and 17.6% from day 0 to 14, 14 to 27, and 27 to 36, respectively (*P* < 0.001). The addition of xylanase partially mitigated the effect of insoluble on feed efficiency from day 14 to 27 and 27 to 36, improving G:F over HF by 13.8% and 8.8%, respectively (*P* < 0.05).

### Energy excretion and dietary energy

By design, GE intake did not differ among treatments (Table 4; *P* = 0.901) Compared with LF, pigs fed HF excreted in total 0.81 more Mcal per day (*P* < 0.001), but the addition of xylanase partially mitigated that effect, as HF + XY excreted 0.17 fewer Mcal per day than HF (*P* < 0.05). When energy excretion was partitioned into fecal and urinary energy, LF excreted the least fecal energy among treatments, and comparatively, the addition of 30% corn bran without solubles increased fecal energy excretion by 207% (*P* < 0.001). The addition of xylanase to HF reduced fecal energy excretion by 7.3% (*P* < 0.05) but increased urinary energy excretion by 9.1%, relative to HF (*P* < 0.05).

**Table 4.** Effect of treatment^1^ on energy balance and digestible, metabolizable, and estimated net energy

	Diet^2^					
Item	LF	HF	HF + XY	HF + AX	Pooled SEM	*P-*value
Energy balance, GE Mcal/d						
Intake	8.14	8.11	8.14	8.13	0.02	0.898
Total excreted	1.44^a^	2.25^b^	2.08^c^	2.32^b^	0.09	<0.001
Fecal	0.88^a^	1.83^b^	1.70^c^	1.93^b^	0.07	<0.001
Urinary	0.56^a^	0.39^b^	0.43^c^	0.39^b^	0.03	0.006
Energy Mcal/kg of DM						
GE	4.34	4.34	4.35	4.34	—	—
DE	3.86^a^	3.35^b^	3.50^c^	3.30^b^	0.05	<0.001
ME	3.56^a^	3.14^b^	3.26^c^	3.11^b^	0.04	<0.001
NE	2.67^a^	2.25^b^	2.35^c^	2.24^b^	0.02	<0.001

^1^ *n* = 15 pigs per treatment fed experimental diets for a 36-d adaptation period followed by a 10-d metabolism crate study. ^2^LF, low-fiber control; HF, high-fiber control; HF+XY, high-fiber control containing 100 mg xylanase/kg; HF+AX, high-fiber control containing 50 mg arabinoxylanoligosaccharide/kg. ^a–c^Within a row, means without a common superscript differ (*P* ≤ 0.05).

The addition of 30% corn bran without solubles increased NDF and ADF content of the HF by 187% and 128% compared with the LF control, respectively (Table 2). Furthermore, when compared with LF, the addition of 30% corn bran without solubles decreased DE by 0.51 Mcal/kg of DM and ME by 0.42 Mcal/kg of DM (*P* < 0.01). However, the addition of xylanase to HF improved DE and ME by 0.15 and 0.12 Mcal/kg of DM, respectively (*P* < 0.05). Although calculated from ME, as expected, LF has the greatest estimated NE, and the addition of xylanase to HF improved estimated NE by 112 Mcal/kg of DM (*P* < 0.05).

### Oxidative status and markers of intestinal integrity and morphology

Pigs fed HF + XY had the greatest serum TAC among treatments (*P* = 0.047) and tended to have less circulating MDA (*P* = 0.098). Similarly, within the distal ileum, pigs fed HF + XY had greater TAC (*P* = 0.037), but MDA concentration within the ileum did not differ among treatments (*P* = 0.108). Although the TAC of the proximal colon did not differ among treatments (*P* = 0.432), pigs fed increased insoluble fiber had reduced MDA in the colon (*P* = 0.041).

Plasma LBP did not differ among treatments (Table 6; *P* = 0.846). Relative to LF, pigs fed increased insoluble fiber had reduced villus height in the ileum (*P* < 0.001). Interestingly, HF + XY and HF + AX had shorter intestinal crypts in the ileum, and HF had the longest crypt, with LF performing intermediately among them (*P* < 0.001). This resulted in pigs fed LF having the greatest V:C ratio, HF having the lowest, and HF + XY and HF + AX performing intermediately among them (*P* < 0.001). Among all treatments, pigs fed xylanase had the greatest mRNA abundance of claudin-4 and occludin in the ileum and tended to have a greater mRNA abundance of zona occludin-1 (*P* = 0.027, 0.047, 0.087, respectively).

**Table 5.** Effect of treatment^1^ on biomarkers of systemic oxidative status and oxidative status of the gastrointestinal tract

	Diet^2^					
Item	LF	HF	HF + XY	HF + AX	Pooled SEM	Treatment *P-*value
Serum						
TAC^3^, mM	4.26^a^	4.35^a^	6.89^b^	4.73^a^	1.15	0.047
MDA,μmol/μL	15.12	16.38	13.26	16.79	1.94	0.098
Ileal tissue						
TAC, mM	2.31^a^	2.52^a^	3.35^b^	2.51^a^	0.15	0.037
MDA, μmol/mg	3.55	2.38	2.64	1.89	0.32	0.108
Colonic tissue						
TAC, mM	5.34	5.81	5.87	5.79	0.27	0.432
MDA, μmol/mg	4.27^a^	2.86^b^	3.18^b^	2.40^b^	0.80	0.041

^1^ *n* = 15 pigs per treatment fed experimental diets for a 36-d adaptation period followed by a 10-d metabolism crate study. ^2^LF, low-fiber control; HF, high-fiber control; HF+XY, high-fiber control containing 100 mg xylanase/kg; HF+AX, high-fiber control containing 50 mg arabinoxylanoligosaccharide/kg. ^3^TAC was measured as a sample’s ability to inhibit the oxidation of 2,2′-Azino-di-3-ethylbenzothiazoline sulfonate and quantified as millimolar Trolox equivalents. ^a, b^Within a row, means without a common superscript differ (*P* ≤ 0.05).

**Table 6.** Effect of treatment^1^ on markers of gut integrity and intestinal morphology

	Diet^2^					
Item	LF	HF	HF + XY	HF + AX	Pooled SEM	*P-*value
Plasma LBP, μg/mL	20.6	22.5	20.8	21.7	1.7	0.846
Intestinal morphology						
Villus height, µm	432.0^a^	403.1^b^	401.1^b^	395.2^b^	4.4	<0.001
Crypt depth, µm	278.7^a^	303.3^b^	269.3^c^	268.2^c^	4.7	<0.001
Villi height: crypt depth	1.6^a^	1.3^b^	1.5^c^	1.5^c^	0.02	<0.001
Gene expression						
*Claudin 3*	1.41	0.93	2.08	0.65	0.56	0.693
*Claudin 4*	0.64^a^	0.52^a^	1.85^b^	0.53^a^	0.36	0.027
*Occuldin*	0.52^a^	0.76^a^	1.27^b^	0.71^a^	0.23	0.047
*Zonula occludens-1*	0.81	1.06	1.21	1.03	0.19	0.087

^1^ *n* = 15 pigs per treatment fed experimental diets for a 36-d adaptation period followed by a 10-d metabolism crate study. ^2^LF, low-fiber control; HF, high-fiber control; HF+XY, high-fiber control containing 100 mg xylanase/kg; HF+AX, high-fiber control containing 50 mg arabinoxylanoligosaccharide/kg. ^a–c^Within a row, means without a common superscript differ (*P* ≤ 0.05).

## Discussion

Non-starch polysaccharides (**NSP**) are often considered an antinutritional factor in swine diets associated with reduced energy and nutrient utilization, decreased growth performance, and reduced carcass yield (Weber et al., 2015; Acosta et al., 2020). Approximately, 49% of the NSP in corn and corn distiller’s dried grains (**DDGS**), primary sources of fiber in many U.S. swine diets, are arabinoxylans (Jaworski et al., 2015). Corn-based arabinoxylans are poorly fermented by the pig due to their low solubility, lignin cross-bridges, and increased amount of l-arabinofuranosyl side chains (Bach Knudsen, 2014). The addition of xylanase to the diet is one strategy considered by nutritionists to mitigate the impact of NSP on pig performance. In poultry, xylanase has been consistently effective in attenuating the antinutritive effects of NSP (Raza et al., 2019), but in pigs, responses are variable, particularly in corn-based diets (Jones et al., 2015; Abelilla and Stein, 2019; Petry et al., 2019). The reason for these varying responses is largely unknown but may be attributed to the length of supplementation, fiber type, and concentration (Patience and Petry, 2019).

In this study, the addition of xylanase improved ADG and G:F in pigs fed a diet high in insoluble corn-based fiber, but these improvements were not detectable until between days 14 and 27 of supplementation and were even greater after day 27. This is in agreement with Lan et al. (2017) who reported that xylanase improved the ADG of nursery pigs fed a corn–soybean meal-based diet from 15 to 28 d of supplementation and improved ADG and G:F from day 29 to 42, and with Myers and Patience (2014) who reported a tendency for xylanase to improve ADG in nursery pigs fed corn-based diets from day 23 to 28 of supplementation. Likewise, Fang et al. (2007) reported improvements in final BW, ADG, and feed efficiency of growing pigs fed a corn–soybean–rapeseed meal-based diet for 49 d. However, there is a paucity of studies that report improved pig performance when xylanase is supplemented in diets containing corn DDGS, and this has been largely attributed to the level of NDF within the diet, and the composition of corn arabinoxylan (Jacela et al., 2010; Kerr et al., 2013). However, data reported herein found xylanase did improve the growth performance of pigs fed a diet with greater than 20% NDF that was predominately supplied by corn-based arabinoxylan. The recalcitrant nature of corn DDGS to xylanase hydrolyzation could be a result of arabinoxylan modification during DDGS production, such as the removal of more degradable arabinoxylans, or an increased ratio of arabinose to xylose (Pedersen et al., 2014; Jaworski et al., 2015). In contrast, corn bran without solubles is a pericarp-enriched dry milled coproduct that is unexposed to fermentation and has a lower arabinose to xylose ratio in both the soluble and insoluble NSP fractions; it is potentially more susceptible to xylanase (Rose et al., 2010; Jaworski et al., 2015).

The time by treatment interaction observed for ADG and G:F may be the result of a shift in fiber digestion to a more efficient digestion site, improved xylose retention by the pig, or increased fiber fermentation by gastrointestinal microbiota, all of which have been shown to improve with increased adaptation time (Castillo et al., 2007; Huntley and Patience, 2018; Petry et al., 2020). Petry et al. (2020) reported that 25 d of adaptation was required for xylanase to improve fiber and energy digestibility in the upper small intestine of growing pigs fed insoluble corn fiber, but responses across the total tract were observed after 7 d of adaptation. Likewise, Huntley and Patience (2018) reported improved xylose, a potential release product of xylanase, retention in pigs with increasing adaptation time. Potentially, if increasing the adaptation time of xylanase shifts fiber digestion into the small intestine, this may promote intestinal absorption of xylose and arabinose, and partially mitigate the losses of energy due to microbial fermentation. This could partially explain the improvements in ADG, G:F, and ME observed, but it is unclear if the metabolic efficiency of xylose would be greater as a monosaccharide or if it was fermented to a volatile fatty acid (Verstegen et al.,1997; Huntley and Patience, 2018). The increased urinary GE excretion observed in HF + XY suggests that some xylose was likely absorbed as a monosaccharide as it has been shown that free xylose increases urine GE due to the excretion of xylose or threitol (Huntley and Patience, 2018).

There are reports of xylanase improving the fermentation of corn-based NSP in vitro, in poultry, and in nursery pigs (Kiarie et al., 2014; Tiwari et al., 2018). Moreover, xylanase may release Stimbiotic arabinoxylan-oligosaccharides (**AX**), that in poultry upregulate fiber-degrading microbial communities and increase the fermentation capacity of the large intestine (Bedford, 2018). Indeed, a study by Zhang et al. (2018) found that supplementing xylanase in a corn-based diet to pigs altered cecal microbiota to favor Firmicutes, and many species within this phylum produce the accessory enzymes to metabolize AX (Sheridan et al., 2016). Conversely, supplementing AX directly herein did not elicit a similar response to xylanase. It is plausible that the AX supplemented in HF + AX was largely fermented in the small intestine and unable to modulate hindgut microbiota, which would explain its lack of effect in this study. On the contrary, xylanase has the potential to release AX throughout the gastrointestinal tract and as such continually modulate the microbiota. It is likely a combination of xylanase improving the digestibility of NSP in the foregut and increasing hindgut fermentation that resulted in improved feed efficiency with increasing adaptation time in this study. Further research is needed to determine the impact of adaptation time on xylanase efficacy to improve NSP digestibility in pigs, if xylanase produces AX in situ from corn-based fiber, and the efficacy of xylanase to modulate gastrointestinal microbiota in pigs.

Although urinary energy excretion increased with xylanase supplementation, it did not outweigh the efficacy of xylanase to improve dietary ME, largely due to the magnitude of improvement in DE. It is well known that pigs do not secrete enzymes capable of degrading NSP and that increasing NSP dilutes dietary energy subsequently decreasing the growth and feed efficiency, as confirmed by reduced growth and increased energy excretion when comparing LF to HF in this study. The addition of xylanase partially improved the energetic contribution of fiber, and this was likely through one or more of the following means: absorption or fermentation of carbohydrate fragments hydrolyzed from arabinoxylan (Adeola and Cowieson, 2011), degradation of the physical fiber matrix resulting in a release of trapped nutrients improving their access to endogenous digestive enzymes (De Lange et al., 2010), mitigation of physiochemical properties associated with NSP that can negatively impact nutrient and energy digestibility (De Vries et al., 2012), or a reduction in the maintenance energy requirement associated with high-fiber diets (Agyekum and Nyachoti, 2017). Indeed, any of the aforementioned xylanase modes of action could explain the improvements in DE, ME, and estimated NE in this study. However, it is unclear which mode of action, or combination of modes of action, is involved in pigs fed corn-based fiber, as the majority of the mechanistic work for xylanase has been conducted in wheat-based diets, in vitro, or in poultry (Bedford, 2018).

Interestingly, supplementing xylanase in this study improved the antioxidant capacity both systemically and within the ileum and decreased markers of systemic lipid peroxidation. Durate et al. (2019) found that xylanase supplementation in nursery diets with corn DDGS reduced MDA in the mucosa of the jejunum and tended to decrease the concentration of protein carbonyls, but Tiwari et al. (2018) found no impact of xylanase on systemic or intestinal markers of oxidative stress in a similar age of pig and diet type. It is unclear how xylanase could decrease oxidative stress and improve the antioxidant capacity of the pig, but one potential mechanism could be through improving the bioavailability of phenolic compounds within the arabinoxylan structure that could serve as antioxidants. Corn arabinoxylan is highly substituted with phenolic compounds derived from hydroxycinnamic acid: caffeic acid, chlorogenic acid, sinapic acid, ferulic acid, and *p*-coumaric acid (Boz, 2015). Of these, ferulic acid is the most abundant and is five times more concentrated in corn bran compared with other cereal brans (Boz, 2015). Ferulic acid is a strong antioxidant capable of scavenging free radicals, stimulating anti-oxidase production, and inhibiting enzymes that cause excess free radical production (Ogiwara et al., 2002), but cereal grain ferulic acid bioavailability is poor due to its esterification within arabinoxylan (Anson et al., 2009). It is possible that xylanase improves ferulic acid bioavailability through the fragmentation of arabinoxylan, thus increasing the access of esterified ferulic acid to ferulic acid esterase produced by the microbiota (Mathew and Abraham, 2004). Furthermore, in vitro production of free ferulic acid, diferulic acid, and ferulic acid–arabinose complexes are increased when xylanase is supplemented in conjunction with ferulic acid esterase, and these products can be passively absorbed by enterocytes and enter the circulatory system (Andreasen et al., 2001; Ou and Sun, 2014). However, it has been suggested that the increased substitution of ferulic acid found in corn-based fiber reduces its susceptibility to fiber-degrading enzymes and fermentation (Pedersen et al., 2014). Further research is warranted if it is found that xylanase does indeed improve the bioavailability of phenolic compounds derived from arabinoxylan in the pig.

The aforementioned improvement in antioxidant status could also potentially explain the increased V:C ratio and mRNA abundance of claudin-4 and occludin observed in HF + XY. The gastrointestinal tract generates free radicals as a byproduct of normal cellular metabolism and certain microbial pathogens can induce oxidative stress in the gastrointestinal mucosa, potentially damaging epithelial cells and affecting gut barrier integrity (Lee et al., 2016; Celi et al., 2017). The antioxidant-increasing effect of xylanase within the ileum may help protect epithelial cells from oxidative damage through direct mitigation of reactive species, reducing cell turnover and improving gut barrier integrity, as supported by the improvements in gut barrier integrity makers observed herein. Others have also reported improvements in markers of enhanced gastrointestinal barrier integrity when xylanase is supplemented alone or as part of an enzyme blend (Tiwari et al., 2018; Li et al., 2018). In totality, the improvements in antioxidant capacity, reductions in oxidative stress markers, and enhanced gut barrier integrity observed in HF + XY may help explain why it is often observed that xylanase supplementation reduces mortality and morbidity in grow-finish pig production.

In conclusion, as dietary fiber increased, the energy within the diet decreased, and this resulted in decreased pig performance, increased energy excretion, and reduced DE and ME. Supplementing xylanase, but not AX, partially mitigated the antinutritive effect of corn-based fiber on performance and dietary energy but appeared to require a longer adaptation time than what is typically reported in the literature. The efficacy of xylanase reported herein also indicates that xylanase is indeed effective in high corn fiber-based diets, and the lack of responses reported when xylanase is supplemented with corn DDGS may not be a result of the concentration of arabinoxylan in corn DDGS-based diets but rather due to other attributes of the arabinoxylan. Moreover, xylanase supplementation in this study provided unexpected health benefits through improving antioxidant capacity, reducing oxidative stress, and enhancing gut barrier integrity. These data indicate that xylanase could potentially improve the utilization of insoluble corn-based fiber, aid in free radical mitigation, and improve gut health, but further research is warranted to elicit the mode of action by which xylanase could improve antioxidant status and gut barrier integrity in the pig.

## Abbreviations

ADF: acid detergent fiber
ADFI: average daily feed intake
ADG: average daily gain
aEE: acid-hydrolyzed ether extract
AX: arabinoxylan-oligosaccharides
BW: body weight
cDNA: complementary deoxyribose nucleic acid
CP: crude protein
Ct: cycle threshold
DDGS: dried distiller grains with soluble
DE: digestible energy
DM: dry matter
G:F: feed efficiency
GE: gross energy
LBP: lipopolysaccharide-binding protein
MDA: malondialdehyde
ME: metabolizable energy
mRNA: messenger RNA
NDF: neutral detergent fiber
NE: net energy
NSP: non-starch polysaccharides
RNA: ribonucleic acid
SID: standard ileal digestible
STTD: standardized total tract digestible
TAC: total antioxidant capacity
TSAA: total sulfur amino acids (Met + Cys)
V:C: villus height to crypt depth
